# Experience-dependent hippocampal pattern differentiation prevents interference during subsequent learning

**DOI:** 10.1038/ncomms11066

**Published:** 2016-03-24

**Authors:** Serra E. Favila, Avi J. H. Chanales, Brice A. Kuhl

**Affiliations:** 1 Department of Psychology, New York University, 6 Washington Pl., New York, New York 10003, USA; 2 Department of Psychology, University of Oregon, Lewis Integrative Science Building, Eugene, Oregon 97403, USA

## Abstract

The hippocampus is believed to reduce memory interference by disambiguating neural representations of similar events. However, there is limited empirical evidence linking representational overlap in the hippocampus to memory interference. Likewise, it is not fully understood how learning influences overlap among hippocampal representations. Using pattern-based fMRI analyses, we tested for a bidirectional relationship between memory overlap in the human hippocampus and learning. First, we show that learning drives hippocampal representations of similar events apart from one another. These changes are not explained by task demands to discriminate similar stimuli and are fully absent in visual cortical areas that feed into the hippocampus. Second, we show that lower representational overlap in the hippocampus benefits subsequent learning by preventing interference between similar memories. These findings reveal targeted experience-dependent changes in hippocampal representations of similar events and provide a critical link between memory overlap in the hippocampus and behavioural expressions of memory interference.

## Abstract (web summary)

There is limited evidence linking learning related changes in hippocampal representations and memory interference. Here Favila and colleagues demonstrate that learning reduces overlap in hippocampal activity patterns corresponding to similar events, which benefits subsequent learning by preventing interference.

## Graphical abstract

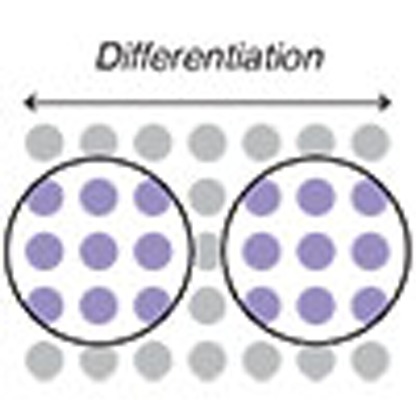

Many of the memories we accumulate share similar features. This similarity can lead to interference or confusability during memory retrieval. Computational models of memory propose that the hippocampus plays a critical role in minimizing the overlap of similar events such that retrieval interference is avoided^1^,^2^,^3^,^4^,^5^,^6^. However, the relationship between event overlap in the hippocampus and learning is potentially bidirectional: lower overlap should prevent interference^1^,^2^,^3^,^4^,^5^,^6^, but learning and behavioural experience may also reduce overlap^7^. Surprisingly, there is limited evidence directly linking the overlap of hippocampal memory representations to behavioural expressions of memory interference; likewise, it is not well understood how, when or why learning reduces overlap of hippocampal representations.

Across rodent and human studies, there are many demonstrations of hippocampal activity patterns changing as a function of learning and behavioural experience^8^,^9^,^10^,^11^,^12^,^13^,^14^,^15^,^16^,^17^,^18^. Intuitively, these experience-dependent changes might reflect discrimination demands or event outcomes. That is, if *A* and *A*′ are similar events that predict the same outcome or require the same behaviour, their representations should converge over the course of learning because differences between the events are uninformative^7^. However, if *A* and *A*′ predict different outcomes or require different behaviour, their representations should diverge with learning^7^. Although there are examples of learning-related divergence of hippocampal representations in the absence of explicit discrimination demands—suggesting that discrimination demands may not fully account for learning-related changes^11^—targeted manipulations are required to gain a clear understanding of whether or how behavioural discrimination demands influence the overlap of hippocampal representations.

To the extent that hippocampal representations diverge with learning, there may be multiple mechanisms that underlie these changes. The mechanism most frequently associated with reducing memory overlap is pattern separation. Pattern separation refers to an orthogonalization of hippocampal activity patterns that is thought to occur automatically and rapidly during event encoding^2^,^5^,^19^,^20^,^21^,^22^,^23^,^24^. Importantly, pattern separation is believed to reduce global similarity among all event representations, such that perfect separation of *A* and *A*′ would render their representations orthogonal: no more similar to each other than to other events (e.g., events *B* and *C*). However, event representations may also continue to diverge as a result of a more targeted differentiation process that pushes similar events apart from one another in representational space by eliminating shared features^25^. Putatively, differentiation is an adaptive response to the coactivation of similar memories during learning—that is, differentiation is triggered by similar memories encroaching on one another during learning^25^. Because differentiation refers to very targeted representational changes, it has the potential to produce more dramatic reductions in memory overlap than would occur via pattern separation. Namely, if *A* and *A*′ are sufficiently differentiated, their representations may become less similar to each other than to events *B* and *C*. As such, differentiation can push representations past the point of orthogonalization.

Regardless of the mechanism that reduces overlap among hippocampal representations, the functional consequence of reduced overlap is thought to be that it protects memories from interfering with one another. Several findings provide indirect support for this idea. For example, rodent hippocampal neurogenesis, which is thought to be critical for pattern separation, is necessary^26^ and sufficient^27^ for discriminating similar spatial contexts. Similarly, hippocampal NMDA receptors are necessary for both pattern separation and spatial context discrimination^22^. Age-related memory decline in rodents^28^ and humans^29^ has also been linked to reduced hippocampal pattern separation. In functional magnetic resonance imaging (fMRI) studies of healthy adults, dissimilarity of hippocampal activity patterns has been correlated with successful remembering^25^,^30^. However, prior studies have not provided direct evidence for a relationship between the overlap of individual hippocampal representations and behavioural expressions of interference between those memories. Thus, to the extent that learning reduces the overlap of hippocampal representations, a critical validation of these changes is that they prevent memory interference.

In the current experiment, we tested for this putative bidirectional relationship between hippocampal representational overlap and learning in humans. Subjects performed a multi-day associative learning task that included pairs of highly similar visual stimuli (images of scenes). Some scene pairs were associated with distinct outcomes (different face images), requiring subjects to discriminate between the similar scenes to recall the associated face, whereas other scene pairs were associated with the same outcome (same face images). In a third, baseline condition scene pairs were exposed to subjects in a task that did not involve any associative learning. We used fMRI multi-voxel pattern analyses to test whether and how associative learning changed the overlap of scene pair representations in the hippocampus. Subjects then performed a new associative learning task with the same scene stimuli, allowing us to test whether learning-related changes in the overlap of hippocampal representations influenced subsequent memory interference between similar scenes. We show that associative learning drives hippocampal representations of similar stimuli away from one another, and that these reductions in representational similarity are associated with reduced interference during later learning. These findings reveal experience-dependent hippocampal processes that adaptively differentiate similar events such that memory interference is avoided.

## Results

**Figure 1:**
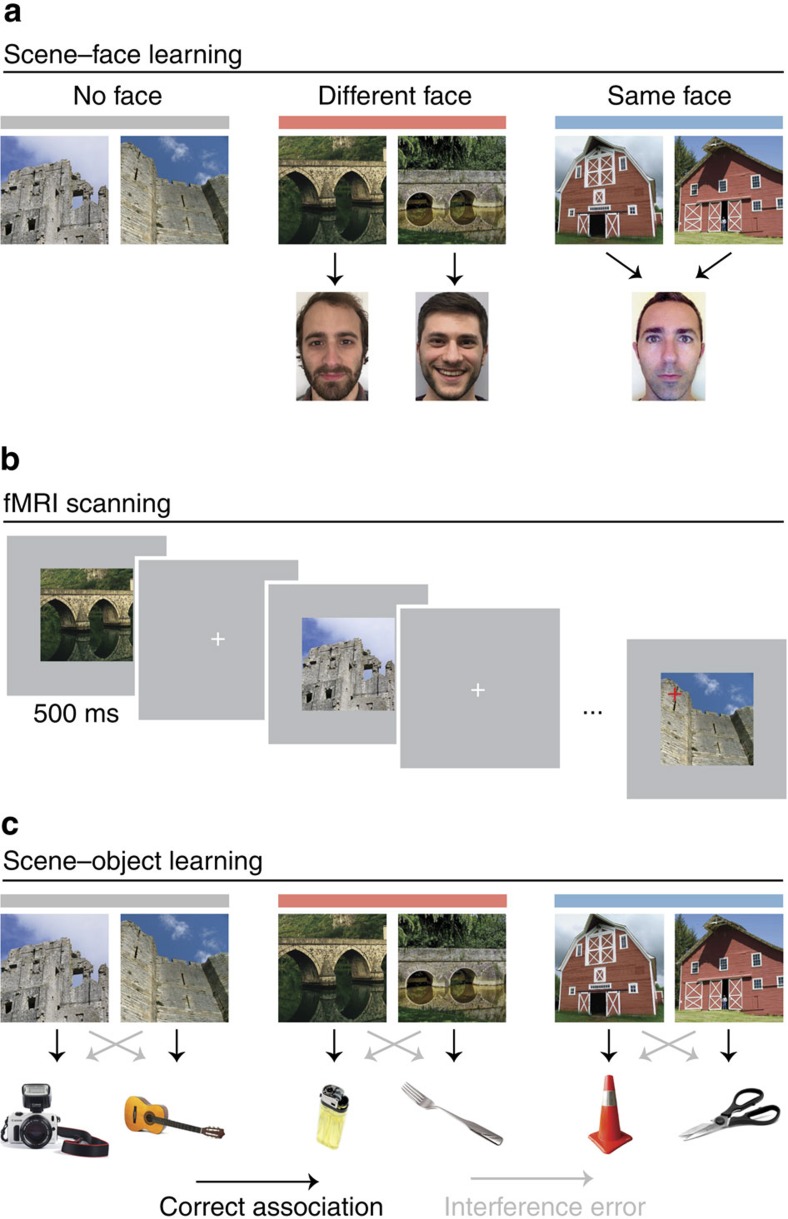
Experimental design. (**a**) On day 1, subjects learned scene–face associations that were constructed using pairs of similar scenes (pairmates). Pairmates were associated with: no image at all (No Face condition), different faces (Different Face condition), or a common face (Same Face condition). Note that pairmates are shown side-by-side to illustrate the design, but subjects never saw any of the scene pairmates simultaneously. (**b**) During fMRI scanning on day 2, subjects viewed the scenes while performing an orthogonal visual target detection task. (**c**) After scanning, subjects learned scene–object associations where each scene was paired with a unique object. Memory for these associations was probed using a forced choice associative memory test. Interference errors occurred when subjects selected an object that had been paired with the scene's pairmate (indicated by grey arrows). See also Supplementary Fig. 1 and Methods section.

Human subjects performed a learning task spanning 2 days (Fig. 1, Supplementary Fig. 1 and Methods section). On day 1, subjects learned 48 scene–face associations through repeated study–test cycles. To create interference, the 48 scenes comprised 24 pairs of perceptually and semantically similar scenes (‘pairmates'; Fig. 1a). For half of the scene pairs, pairmates were associated with different faces (Different Face condition), creating a demand to discriminate between similar scenes (so as to retrieve the correct face). For the other half of pairs, pairmates were associated with the same face (Same Face condition), meaning that there was no behavioural demand to discriminate between the pairmates. An additional 24 scenes (12 pairs) were viewed by subjects on day 1 in an exposure task with no associated faces or learning requirements (No Face condition; Fig. 1a and Supplementary Fig. 1a). None of the scene pairmates in any condition were ever shown simultaneously, meaning any discrimination between pairmates was necessarily memory based.

On day 2, subjects repeated an abbreviated training session and then entered the fMRI scanner. During scanning, subjects viewed the scenes from all conditions (Different Face, Same Face, No Face) many times while performing a cover task of detecting infrequent red crosses (Fig. 1b). fMRI data were used to estimate the pattern of neural response to each scene, allowing us to test whether prior learning influenced representational overlap between scene pairmates. After scanning, subjects completed three study–test cycles of a scene–object learning task where all of the scenes presented during fMRI scanning were paired with a unique and novel object (Fig. 1c and Supplementary Fig. 1b). On each scene–object test trial, subjects were presented with a scene and three object choices: the target object (correct), the object that was associated with the scene's pairmate (‘pairmate foil'; interference error) and an object that was associated with a nonpairmate scene (‘nonpairmate foil'; other error).

### Behaviour

Subjects completed a minimum of six scene–face test rounds (mean=7.78) on day 1 and a minimum of two rounds on day 2 (mean=2.22). Subjects were required to reach 100% accuracy on both days. During fMRI scanning, subjects indicated whether visual targets (red crosses) were present or absent with high accuracy (mean=97.25%).

We used mixed-effects logistic regression to test whether scene–object memory was influenced by prior scene–face learning condition. Accuracy on each scene–object trial was modelled as a function of scene–face condition, scene–object test repetition (1–3), and scene subcategory (indoor versus outdoor). There was a significant main effect of scene–face condition (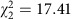, *P*<0.001; Supplementary Fig. 2a). Linear contrasts revealed facilitation in scene–object memory for scenes from the Different Face condition versus the Same Face (*β*=1.12, s.e.=0.26, *z*=4.27, *P*<0.001) and No Face conditions (*β*=0.91, s.e.=0.22, *z*=4.07, *P*<0.001). Thus, at a behavioural level, the demand to discriminate similar scenes during scene–face learning benefitted later scene–object learning. There was also a significant main effect of test repetition (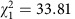, *P*<0.001), with accuracy increasing across the three scene–object test cycles (*β*=1.20, s.e.=0.14). There was no main effect of scene subcategory (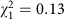, *P*=0.72). Notably, subjects committed interference errors more often than other errors during scene–object learning (*F*_1,17_=33.12, *P*<0.001; Supplementary Fig. 2b), confirming that our paradigm was effective at inducing interference.

### Learning reduces overlap of hippocampal representations

**Figure 2:**
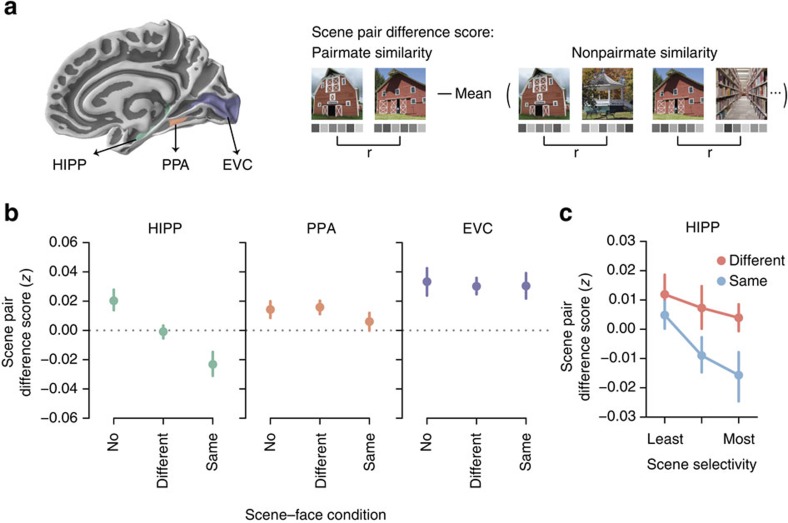
Learning reduces representational overlap in the hippocampus. (**a**) ROIs included scene-preferring voxels in hippocampus (HIPP), parahippocampal place area (PPA) and early visual cortex (EVC). Note: we use ‘HIPP' to specifically refer to the ROI we used, and not to the hippocampus in general. ROIs are displayed on the right hemisphere of the Freesurfer average cortical surface in the Figure, but were defined bilaterally in each subject's native space for all analyses. For each subject, a neural activity pattern was estimated for each scene. Neural similarity was operationalized as Fisher *z*-transformed Pearson's correlations between scene patterns. For each scene pair, a scene pair difference score was computed by subtracting the mean within-condition nonpairmate similarity from the pairmate similarity. This difference score reflected the relative representational distance between pairmates. (**b**) Prior learning (Different Face and Same Face conditions) decreased representational overlap between pairmates (lower scene pair difference scores) in HIPP (main effect of scene-face condition: *F*_2,34_=7.75, *P*=0.0017), but not in PPA (Friedman 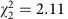, *P*=0.35) or EVC (*F*_2,34_=0.074, *P*=0.93). (**c**) For the Different and Same Face conditions, decreased representational overlap (lower scene pair difference score) was most evident in voxels with the greatest scene selectivity (*F*_2,34_=3.98, *P*=0.028). Plotted data represent mean±s.e.m. across subjects. See also Supplementary Fig. 3.

Next, we considered whether scene–face learning influenced the representational overlap of scene pairmates. To compute representational overlap, we correlated each scene's multi-voxel activity pattern (see Methods section) with the activity pattern for every other scene in the same condition, including the scene's pairmate. As a validation of our measure's sensitivity, we expected scene pairmates to be associated with greater neural similarity than nonpairmates. For example, ‘castle 1' and ‘castle 2' should have more overlapping neural activity patterns than ‘castle 1' and other scenes (Fig. 1a). We considered three regions of interest (ROIs; Fig. 2a): scene-preferring voxels within the hippocampus (HIPP), scene-preferring voxels in medial ventral temporal cortex (parahippocampal place area; PPA), and early visual cortex (EVC). Voxelwise scene preference was evaluated using data from an independent category localizer scan (see Methods section).

In the No Face condition, pairmate similarity was significantly greater than nonpairmate similarity in EVC (*t*_17_=3.40, *P*=0.0034), PPA (*t*_17_=2.30, *P*=0.034) and HIPP (*t*_17_=2.64, *P*=0.017). Importantly, this indicates that each of our ROIs exhibited baseline sensitivity to the perceptual and/or semantic similarity of pairmates. For subsequent analyses, we focus on the difference between pairmate and nonpairmate similarity (hereinafter, scene pair difference score) as a critical measure of the representational structure of scenes (Fig. 2a). Note that if pairmates were orthogonally coded—coded as if they were two unrelated scenes—this would correspond to a scene pair difference score of 0.

**Figure 3:**
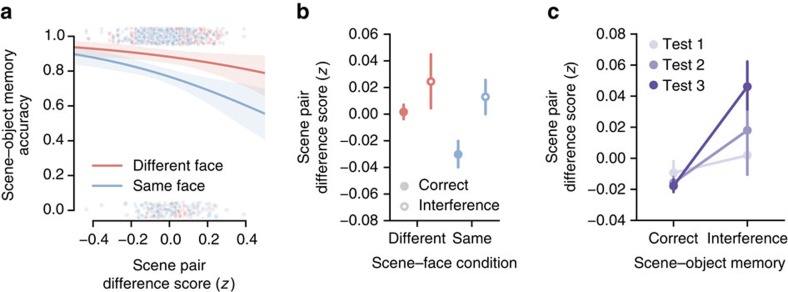
Learning-driven decreases in representational overlap prevent interference during future learning. (**a**) In a logistic regression analysis, lower hippocampal scene pair differences scores (lower representational overlap) in the Same Face and Different Face conditions predicted resistance to interference during scene-object learning (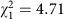, *P*=0.030). Each point represents an individual scene pair difference score from scene-preferring hippocampal voxels pooled across subjects. Lines and shading indicate logistic regression fits and standard errors for each condition. (**b**) As an alternative visualization of **a**, mean scene pair difference scores are shown as a function of scene-face condition (Same Face, Different Face) and subsequent scene-object memory (correct versus interference). Data are averaged across scene–object test repetition. (**c**) Scene pair difference score interacted with test repetition in predicting subsequent scene-object memory (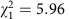, *P*=0.015): scene-object interference errors in later test repetitions were associated with larger scene pair difference scores than errors in early test repetitions. Scene pair differences scores are averaged across the Same Face and Different Face conditions for each test repetition. For **b** and ,**c** plotted data represent mean ±s.e.m. across subjects. See also Supplementary Figs 4 and 5.

To test whether scene-face learning influenced scene pair difference scores, separate one-way analysis of variances (ANOVAs) were applied to each ROI. The effect of learning condition on scene pair difference score was highly robust in HIPP (*F*_2,34_=7.75, *P*=0.0017), but absent in EVC (*F*_2,34_=0.074, *P*=0.93) and PPA (Friedman 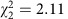, *P*=0.35; Fig. 2b). There was also a significant interaction between ROI and learning condition on scene pair difference score (*F*_4,68_=3.70, *P*=0.0087). In HIPP, scene pair difference scores were significantly lower in the Different Face condition than in the No Face condition (*t*_17_=−2.31, *P*=0.033). Surprisingly, difference scores were also significantly lower in the Same Face condition than in the No Face condition (*t*_17_=−3.79, *P*=0.0015). That is, both learning conditions elicited decreases in the overlap of pairmate representations, relative to nonpairmate representations. If anything, scene pair difference scores were marginally lower in the Same Face condition than the Different Face condition (*t*_17_=−1.82, *P*=0.087) despite the fact that discrimination demands were only present in the Different Face condition. When only considering scene pairs associated with correct (interference-free) scene–object memory, scene pair difference scores in HIPP were significantly lower in the Same Face condition than Different Face condition (*t*_17_=−2.34, *P*=0.032; Fig. 3b). Learning-related decreases in scene pair difference scores reflected a combination of increased similarity among nonpairmates and decreased similarity of pairmates (Supplementary Fig. 3a), indicating that learning had opposing influences on pairmate and nonpairmate similarity.

While hippocampal scene pair difference scores were significantly greater than zero in the No Face condition, this effect was eliminated in the Different Face condition (*t*_17_=−0.18, *P*=0.86) and significantly reversed in the Same Face condition (*t*_17_=−2.55, *P*=0.021). That is, when two similar scenes were paired with the same face, their hippocampal representations became less similar to each other than to nonpairmate scenes. Strikingly, despite this negative scene pair difference score in HIPP, the same stimuli were associated with a significantly positive difference score in EVC (*t*_17_=3.55, *P*=0.0025) and a numerically positive score in PPA (*W*_17_=84.0, *P*=0.96).

To determine whether changes in scene pair difference scores were selective to scene-preferring hippocampal voxels, we repeated the same analysis on hippocampal voxels that varied in scene selectivity. Specifically, for each subject we divided hippocampal voxels into terciles from least to most scene-preferring, based on data from the independent localizer scan. An ANOVA with factors of learning condition (Different Face and Same Face) and scene selectivity of voxels (terciles) revealed a significant main effect of tercile on scene pair difference score (*F*_2,34_=3.98, *P*=0.028), with the largest reduction in scene pair difference score observed in the most scene-preferring hippocampal voxels (Fig. 2c). Thus, scene–face learning preferentially altered representational structure in the hippocampal voxels with the strongest preference for scenes.

### Learning-driven differentiation prevents later interference

Having established that scene–face learning reduced representational overlap of scene pairmates in the hippocampus, the second objective was to test whether lower representational overlap protected memories from interference during subsequent scene–object learning. Interference errors were defined as scene–object test trials where subjects mistakenly matched a given scene with the object that was associated with its pairmate (pairmate foil; Fig. 1c). For example, after learning that ‘castle 1' is paired with ‘camera' and ‘castle 2' is paired with ‘guitar,' an interference error would occur if a subject incorrectly retrieved ‘guitar' when cued with ‘castle 1.' These interference errors constituted the majority of error trials (Supplementary Fig. 2b).

We used mixed-effects logistic regression to test whether scene pair difference scores in the Different and Same Face conditions predicted binary memory outcomes on the scene–object memory test: correct retrieval of the target associates versus interference errors (either 1 or 2 interference errors for a given pair). The full additive model included scene pair difference score, prior scene–face condition, scene–object test repetition and scene subcategory as predictors. Critically, there was a significant main effect of hippocampal scene pair difference score on scene–object memory (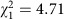, *P*=0.030). Specifically, lower scene pair difference scores predicted reduced interference (higher accuracy) during subsequent scene–object learning (*β*=−2.01, s.e.=0.87; Fig. 3a,b and Supplementary Figs 4 and 5 for related analyses). Importantly, this relationship was absent in EVC (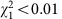, *P*=0.98) and PPA (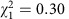, *P*=0.59). Moreover, when considering the raw correlation between pairmates (as opposed to the scene pair difference score), the relationship in HIPP remained significant (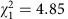, *P*=0.028), whereas raw nonpairmate correlations did not predict accurate memory versus interference errors (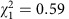, *P*=0.44; Supplementary Fig. 4a).

Because scene–object memory was probed across three test cycles, we also tested whether the relationship between scene pair difference scores and scene–object memory varied across the test session. We added an interaction term between hippocampal scene pair difference score and scene–object test repetition to our model and tested this model against the additive model above. Indeed, there was a significant interaction (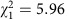, *P*=0.015). Specifically, the relationship between scene pair difference score and subsequent scene–object memory became stronger across test repetitions (*β*=−2.64, s.e.=1.06; Fig. 3c). Pairs associated with interference errors that occurred later in scene–object learning (after more opportunities for study) were characterized by relatively greater representational overlap than those associated with earlier interference errors (Fig. 3c and Supplementary Figs 4b and 5 for related analyses).

## Discussion

Theoretical and computational perspectives have argued that overlap of neural activity patterns in the hippocampus is related to memory interference^1^,^2^,^3^,^4^,^5^,^6^. Nevertheless, there remains surprisingly little evidence directly linking interference between individual memories to the overlap of their hippocampal representations. Moreover, there is no clear understanding of how representational overlap in the hippocampus is influenced by learning experience and behavioural demands. Here using pattern-based fMRI measures of hippocampal memory overlap in humans, we provide data to address both of these open questions. First, we show that associative learning reduced overlap between hippocampal representations of highly similar visual stimuli. Critically, these learning-induced reductions in memory overlap were at least as robust when similar stimuli predicted the same outcome (Same Face condition) as when they predicted different outcomes (Different Face condition), indicating that they were not explained by task demands or predicted outcomes. Finally, learning-induced reductions in representational overlap were adaptive: lower overlap of scene pairmates predicted reduced interference between those stimuli during subsequent associative learning. These findings provide critical support for the argument that overlap among hippocampal representations contributes to memory interference^1^,^2^,^3^,^4^,^5^,^6^, but challenge current ideas concerning how hippocampal representations of overlapping experiences change with learning.

**Figure 4:**
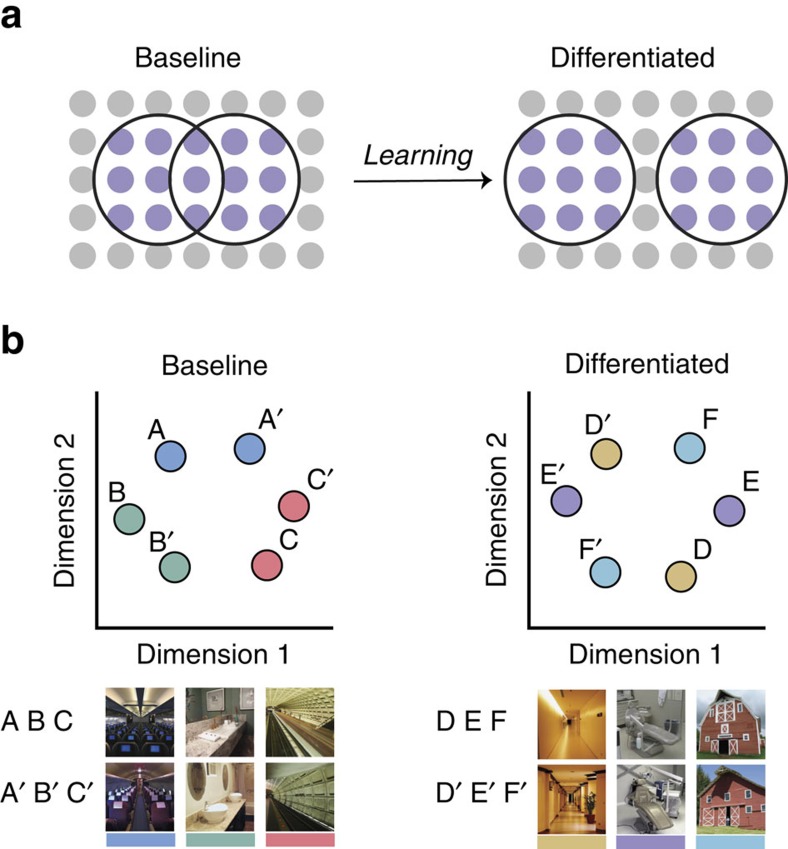
Schematic illustration of hippocampal differentiation. (**a**) Representational overlap between scene pairmates can be conceptualized in terms of shared feature units. Here, each scene representation (black circle) is composed of active units (purple circles). Before learning (that is, in the ‘Baseline' or No Face condition), pairmate representations share common features, as reflected by overlap of the black circles. After learning, however, the pairmate representations diverge, with shared units ‘dropping out' of the representation. (**b**) The representational distance (overlap) between pairmate and nonpairmate representations can be visualized using multidimensional scaling (MDS). Here, neural similarity measures from three No Face scene pairs and three Same Face scene pairs, drawn from a single subject, are projected into a two-dimensional representational space. In the baseline state (No Face condition), the representational distance between pairmates (for example, *A* and *A*′) is lower, on average, than between nonpairmates (for example, *A* and *C*). That is, pairmates are ‘clustered' in representational space. This pairmate clustering corresponds to a positive scene pair difference score. However, learning produces differentiation, pushing pairmate representations apart from one another in representational space and eliminating parimate clustering. After differentiation (Same Face condition), pairmates systematically occupy opposing positions in representational space, resulting in greater representational distances between pairmates than nonpairmates. This corresponds to a negative scene pair difference score.

To characterize how learning-influenced memory overlap, we measured the representational structure derived from neural activity patterns^31^. Specifically, we considered the overlap (correlation) of activity patterns for scene pairmates relative to the overlap of nonpairmates—what we refer to as the scene pair difference score. Focusing on the relative position of pairmates versus nonpairmates in representational space greatly facilitates comparisons across brain regions or conditions that might differ in terms of raw correlation values^32^. For example, although HIPP, PPA and EVC were associated with different mean raw correlation values (Supplementary Fig. 3a,c), across these regions there was a common representational structure in the No Face condition wherein scene pairmates were more similar to each other than to nonpairmates, as would be expected. However, within the hippocampus—and only the hippocamus—this structure changed with learning. Namely, there was a marked decrease in the relative overlap of pairmate representations in the Different and Same Face conditions (lower scene pair difference scores). That is, learning drove pairmates apart from one another in representational space. These changes in representational structure are illustrated with multidimensional scaling in Fig. 4b, using sample data and stimuli from our experiment. As can be seen in the Figure, pairmates are clustered together in representational space in the baseline state (No Face condition). With learning, this clustering is eliminated or reversed, as pairmates are differentiated from one another.

What mechanism accounts for the learning-related changes we observed? One possibility is that, relative to the No Face condition, the Same and Different Face conditions simply demanded greater attention to the scenes. While it is probable that attention to the scenes differed across conditions, our full pattern of results is not easily explained in terms of differences in attention. First, reductions in representational overlap were completely absent in EVC and PPA—regions that are the most likely candidates to reflect modulations of visual attention^33^,^34^. In this regard, the selectivity of our results to the hippocampus is very important. Second, because the Different Face condition required subjects to discriminate between similar scenes, attentional demands should have been higher in the Different Face condition than the Same Face condition. Yet, representational overlap was, if anything, lower in the Same Face condition than the Different Face condition. Finally, an attention-based account does not explain why we observed a negative scene pair difference score in the Same Face condition (a point we elaborate on below).

Comparing results in the Same Face and Different Face conditions also helps to rule out other accounts of our findings. As previously described, if reduced hippocampal overlap was driven by discrimination demands or by the predicted event outcomes (that is, the faces associated with the scenes), we would have expected decreased overlap in the Different Face condition and increased overlap in the Same Face condition^7^,^15^,^35^. While we did observe this pattern of results outside of the hippocampus (Supplementary Fig. 6b), the results in the hippocampus were clearly incompatible with this account. Moreover, when also considering that the reduction in representational overlap in the hippocampus was strongest in voxels that were independently identified as scene preferring, the most parsimonious argument is that representational changes were related to the scene images themselves and not the outcomes (faces) associated with the scenes.

Another critical aspect of our results is that scene pairs in the Same Face condition were associated with negative scene pair difference scores. In other words, hippocampal representations of pairmates became less similar to each other than to nonpairmate scenes. For example, ‘barn 1' became less similar to ‘barn 2' (its pairmate) than to ‘gazebo 2' and ‘library 2' (nonpairmates; Fig. 2a). As illustrated in Fig. 4b, this corresponds to a repulsion of pairmates away from one another in representational space. This paradoxical finding was clearly a result of learning because we observed precisely the opposite effect—a positive scene pair difference score, or pairmate clustering (Fig. 4b)—in the No Face condition. Moreover, it is striking that the Same Face condition yielded a negative scene pair difference score in HIPP despite the scene pair difference score remaining strongly positive in EVC, and numerically positive in PPA. Thus, this flip in representational structure was selective to the hippocampus. Importantly, a negative scene pair difference score is not explained by standard accounts of pattern separation because perfect pattern separation should only produce orthogonalization of activity patterns. For example, if ‘barn 1' and ‘barn 2' were orthogonally coded, they would each be represented as unique scenes, with ‘barn 2' no more similar—and no less similar—to ‘barn 1' than to other scenes. This would produce a scene pair difference score of 0 and would correspond to pairmates being randomly distributed in representational space. Thus, the negative scene pair difference score in the Same Face condition is uniquely consistent with a differentiation account. For this reason, the fact that we considered pairmate similarity relative to nonpairmate similarity was a critical feature of our analysis. To be clear, however, we do not argue that pattern separation was absent in our experiment. Rather, while some degree of pattern separation likely occurred during initial encoding, we argue that the learning-induced reductions in representational overlap that we observed reflect an additive effect of differentiation^25^.

Why would differentiation occur when two scenes predict the same outcome? Putatively, differentiation occurs when two overlapping representations are simultaneously active and compete to establish a representational foothold^25^. Competition of this form can be created by pairing multiple stimuli with a common associate^36^,^37^. Here when two scenes were associated with a common face, this may have increased the probability that presentation of one of the scenes (for example., ‘barn 1') triggered reactivation of the pairmate scene (‘barn 2'). The hippocampus would resolve the competition between the similar scenes by eliminating shared features (Fig. 4a), thereby selectively differentiating the two representations from one another^25^. Although the idea that simultaneous activation triggered differentiation in the present study is speculative, this account can explain our full set of findings and is consistent with existing theoretical models^25^,^37^,^38^ and other recent empirical observations of competition-driven memory weakening^25^,^39^,^40^,^41^.

Our finding of learning-induced hippocampal differentiation is consistent with evidence from a recent study by Schlichting *et al*^18^. In their study, subjects learned associations between pairs of object images, with some pairs containing a common object (for example, an AB pair and a BC pair contain a common B element). fMRI activity patterns corresponding to the individual event elements (A, B, C) were measured pre- and post learning. They found that, within the posterior hippocampus, learning produced a relative differentiation of activity patterns corresponding to A and C elements that shared a common B element. This observation is consistent with our finding of reduced scene pair difference scores in the Same Face condition, providing converging evidence that, at least in some contexts, linking events to a common outcome will paradoxically drive corresponding hippocampal representations apart.

Despite some consistency across studies, our findings and paradigm differ from the study by Schlichting *et al*.^18^ in several important ways. First, here we specifically selected pairs of images with high perceptual and semantic similarity (scene pairmates) and we confirmed, via our post-scan scene–object learning test, that this similarity between pairmates produced memory interference errors. We also showed that in our baseline (No Face) condition, scene pairmates were represented as more similar than nonpairmate scenes in the hippocampus. This baseline representational similarity sharply contrasts with the end-state of learning: either an elimination (the Different Face condition) or a complete reversal of baseline similarity (the Same Face condition). Thus, we specifically show that hippocampal representations of highly similar and confusable events become differentiated with learning. Indeed, the degree of event similarity may be a critical factor in determining the degree of differentiation that occurs^15^. Our learning manipulation also differed from the study by Schlichting *et al*.^18^ in that we directly manipulated behavioural discrimination demands. This manipulation afforded the critical insight that hippocampal overlap decreased as much or more when discrimination demands were absent, relative to present. Finally, as we detail below, a critical and novel aspect of the present study is that we directly related the overlap of individual hippocampal representations to subsequent behavioural expressions of memory interference.

Several findings suggest a critical role for the hippocampus in disambiguating similar memories. For example, prior studies have shown that hippocampal lesions disproportionately impair perceptual discrimination in rodents when displays contain many items^42^, when item locations are similar^43^, or when similar items are introduced before or after learning^44^. Likewise, human patients with medial temporal lobe damage exhibit heightened susceptibility to memory interference^45^,^46^, and fMRI measures of hippocampal activity during learning have been associated with reduced memory interference^47^. A novel element of the present study, however, is that we related interference between individual memories to the overlap of their hippocampal activity patterns. This finding provides critical support for the hypothesis that overlap among hippocampal representations is related to memory interference. Moreover, it provides crucial validation that the learning-induced reductions in representational overlap that we observed carried a functional consequence.

Although we show that hippocampal differentiation predicted memory interference, it is unlikely that it was the only influence on behaviour. Indeed, although representational overlap was lowest in the Same Face condition, behavioural performance was best in the Different Face condition. Thus, a factor other than hippocampal differentiation is required to explain the better performance in the Different Face condition. Post-experiment debriefings revealed that many subjects utilized explicit learning strategies that may have influenced performance. In particular, most subjects (13/18) reported integrating scene-face associations into subsequent scene–object associations. This strategy would be helpful in the Different Face condition because it would add a differentiating element (distinct faces) to the overlapping scene–object associations. However, it would not be helpful in the Same Face condition because it would add a common element (the same face) to the overlapping scene–object associations. As an indirect test of this idea, we probed for face reactivation during the scanned scene-viewing task. Indeed, there was modest evidence for face reactivation within parietal cortex^48^ (Supplementary Fig. 6a,b). Moreover, individual differences in parietal face reactivation were positively related to subsequent scene–object learning in the Different Face condition, but not in the Same Face condition (Supplementary Fig. 7), consistent with our prediction. Ultimately, it is likely that behaviour was determined by multiple factors, but explicit integration of scene–face and scene–object associations is at least one factor that may account for better overall performance in the Different Face condition than the Same Face condition. Importantly, however, this strategy was dissociable from the influence that hippocampal pattern differentiation had on subsequent learning.

In summary, our findings reveal experience-dependent changes in hippocampal representations that specifically exaggerate the difference between similar events. These changes are highly selective to the hippocampus and are triggered by event similarity during learning, not behavioural discrimination demands. Finally, we show that reduced overlap among hippocampal representations protects memories from interference during subsequent learning.

## Methods

### Subjects

Twenty-three right-handed native English speakers (19–30 years old, 6 males) participated in the experiment after giving written informed consent to procedures approved by the New York University Institutional Review Board. Three subjects were withdrawn from the experiment due to discomfort or for failing to comply with instructions. Two more participants were excluded from data analysis due to excessive head motion or sleepiness. This yielded a final data set of 18 subjects. This sample size is consistent with similar fMRI studies in the field and was determined before data collection.

### Stimuli

The learning tasks involved 72 non-famous scene images, 36 non-famous male face images and 72 everyday object images. All images were in colour. The 72 scene images consisted of 36 pairs of perceptually and semantically similar scenes (pairmates), with an equal number of indoor and outdoor pairs. For each subject, 12 scene pairs were randomly assigned to each of three learning conditions (No Face, Different Face and Same Face; Fig. 1), while balancing for scene subcategory within condition. There was no pairmate structure for faces or objects. For each subject, 24 faces were randomly assigned to the scenes from the Different Face condition and the remaining 12 faces to the scene pairs from the Same Face condition. Finally, 24 objects were randomly assigned to the scenes from each learning condition for each subject. Note: all figures contain public domain images representative of the stimuli used in the experiment, but not identical to them. Scene, face and object stimuli used in the experiment can be downloaded at https://dx.doi.org/10.6084/m9.figshare.2087638.

Stimuli for the functional localizer were drawn from a published stimulus set^49^ and consisted of scrambled images and face, scene (house and corridor) and object (car and guitar) images on phase-scrambled backgrounds. All images were greyscale, and face, scene and object images varied in size, position and viewpoint.

### Experimental procedure

The experiment consisted of an associative learning task divided across 2 days. We designed the learning task as a modified version of the AB–AC task commonly used to study memory interference. On day 1, subjects learned 24 Different Face and 24 Same Face scene–face associations to a 100% criterion through interleaved study and test. Subjects were told that their goal was to learn the face that was associated with each scene but were not informed that scenes existed in pairs or that some scenes shared a face associate. During study trials, subjects were shown the scene (1,500 ms) and then its associated face (1,500 ms) after a 500 ms delay. Subjects were instructed to intentionally encode each scene–face pair during study trials, but no responses were required. During test trials, scene cues (1,500 ms) were followed by a set of three faces, from which subjects had 5,000 ms to select the target face. After a response was made or time had elapsed, feedback was provided by showing the target face alone for 1,000 ms. For scenes from the Different Face condition, the three face options included the target face, the pairmate foil (face associated with the cue's pairmate) and a randomly chosen nonpairmate foil (face associated with a nonpairmate scene) from the Different Face condition. For scenes from the Same Face condition, the three face options included the target face, and two randomly chosen nonpairmate foils from the Same Face condition. To start the learning session, half of the Same Face scene pairs and half of the Different Face scene pairs were selected and the corresponding scene–face associations (24 trials) were studied in random order and then tested in random order. The remaining half of the associations were then studied and tested. This constituted the first study–test cycle. A second study–test cycle was completed using an identical procedure, but with a different random grouping of associations into halves. This constituted the second study–test cycle. Nonpairmate foils for test trials were always selected from the set of stimuli presented in the immediately preceding study block. After the two study–test cycles, subjects completed at least four additional test blocks with feedback. In each test block, all scene–face associations (48 trials) were tested in random order. If subjects did not achieve 100% accuracy in the last test block, additional test blocks were completed until 100% accuracy. Thus, on the first day subjects were tested on each association a minimum of six times. It should also be noted that under this procedure, pairmate associations were learned in an interleaved, not blocked, fashion. Separately in an exposure phase, all scenes (including those in the No Face condition) were viewed eight times in a visual target detection task with no learning demands. In this task, scenes appeared on the screen for 500 ms each and subjects indicated as quickly as possible when an inverted scene was presented.

At the start of day 2, subjects were tested on every Same Face and Different Face association at least twice (once per block, as at the end of day 1) and until 100% accuracy was achieved. Subjects then participated in another round of the exposure task, where all scenes (including No Face scenes) were viewed eight times. Importantly, at no point during the scene–face learning or exposure tasks were scene pairmates simultaneously presented—thus, there was never an opportunity for perceptual discrimination.

Next, subjects participated in an fMRI scanning session. In an event-related design, subjects viewed all scenes between 8 and 10 times each while maintaining fixation and performing a cover task of detecting infrequent red crosses (8.33% of trials). Each fMRI run consisted of the 72 scenes as well as 24 null-fixation trials (18 interleaved, 3 lead in, and 3 lead out). Each trial was 4,000 ms long, with the stimulus presented centrally on a grey background for 500 ms followed by a 3,500 ms intertrial interval. Randomly interleaved null trials provided jitter. For stimulus presentation purposes, scene stimuli were divided into two sets of 36 images by splitting all scene pairs and assigning each pairmate to one of two sets. For each run, stimulus presentation order was randomized within set, and the order of the sets was counterbalanced. Thus, every run contained one presentation of every scene stimulus, with scene pairmates always presented in different halves of the run. Presentation orders were not repeated across subjects or runs. Subjects also performed a separate functional localizer task that included 6 s blocks of scene, face, object and scrambled stimuli presented at 2 Hz (ref. 49). Stimuli were displayed on a projector at the back of the scanner bore, which subjects viewed through a mirror attached to the head coil. Subjects made responses on an MR-compatible button box.

After scanning, subjects learned 72 unique scene–object associations by completing three study–test cycles. Trial timing was identical to that of scene–face learning but there was no feedback on test trials. On test trials, subjects were cued with a scene and chose from three objects: the target, the pairmate foil, and a randomly selected nonpairmate foil from the same condition as the scene cue. For all three study–test cycles, the 72 associations were studied and tested in sets of 24 trials, each of which were balanced by condition. For each set of 24 trials, a balanced number of scene pairs from each of the three conditions were randomly selected, and the corresponding associations were studied in random order and then tested in random order. This procedure was identical for study–test cycles two and three, but with different random assignments of stimuli to each set of 24 trials. As in scene–face learning, nonpairmate foils for test trials were always selected from the set of stimuli in the immediately preceding study round.

See also Fig. 1 and Supplementary Fig. 1.

### Image acquisition and preprocessing

We acquired all images on a 3T Siemens Allegra MRI system. Functional data were acquired with a T2*-weighted echo-planar imaging sequence with partial coverage (repetition time=2,000 ms, echo time=30 ms, flip angle=82°, 34 slices, 2.5 × 2.5 × 2.5 mm voxels) and an eight-channel occipital surface coil. We confirmed via pilot fMRI scans that the occipital surface coil yielded enhanced sensitivity extending to posterior medial temporal lobe regions. Oblique coronal slices were aligned perpendicular to the calcarine sulcus at the occipital pole and extended anteriorly covering ventral temporal cortex and the medial temporal lobe. We also acquired a whole-brain high-resolution T1-weighted magnetization-prepared rapid acquisition gradient echo anatomical volume (1 × 1 × 1 mm voxels).

FSL^50^ was used for functional image preprocessing. The first six volumes of each functional run were discarded to allow for T1 stabilization. To correct for head motion, each time series was realigned to its middle volume. The localizer data were spatially smoothed using a 6 mm full-width at half maximum gaussian kernel. Data from the main experiment were not spatially smoothed. All data were high-pass filtered using gaussian-weighted least squares straight line fitting with *σ*=64.0 s. Timepoints with motion relative to the previous volume greater than half the width of a voxel (1.25 mm) were identified and excluded from further analysis. Freesurfer^51^ was used to segment the grey–white matter boundary and construct a model of the cortical surface from the high-resolution anatomical image.

### fMRI general linear model (GLM) analyses

All fMRI analyses were performed in subjects' native space. Using FSL, we conducted a voxelwise analysis of each subject's unsmoothed timeseries data from the main experiment with a model that included a regressor of interest for each of the 72 scene stimuli. These regressors were constructed as impulses and were then convolved with a canonical double-gamma hemodynamic response function. Six realignment parameters were included as regressors of no interest to control for motion confounds. Run-level models were estimated using gaussian least squares with local autocorrelation correction (pre-whitening) and then entered into a fixed-effects model. This procedure produced *t*-maps representing the activation elicited by each scene relative to baseline for each subject. No normalization to a group template was performed.

To analyse the localizer data, we constructed a model with three regressors of interest corresponding to the three visual categories (scene, face, object). These regressors were constructed as boxcar functions onsetting with the first image of a category block and lasting for the duration of the block. Realignment parameters were included and the model was estimated as described above but using smoothed timeseries data. A linear contrast of scenes greater than faces and objects was used to obtain subject-specific estimates of scene sensitivity for each voxel.

### fMRI pattern similarity analyses

Neural similarity between scenes was operationalized as the Fisher *z*-transformed Pearson correlation between *t*-maps. Because scene pairmates were presented in two blocked sets (i.e., a given scene was always presented in a different half of the run than its pairmate; see Experimental procedures), we only computed across-set correlations between our 72 scenes. This produced a 36 × 36 matrix, with the diagonal containing pairmate similarity values and all other cells containing nonpairmate similarity values. Cells corresponding to across-condition nonpairmate similarity values were not analysed. Scene pair difference scores were computed by taking the similarity of two pairmates and subtracting from it the mean similarity of each pairmate to all other nonpairmate scenes from the same condition (Fig. 2a). Thus, scene pair difference scores reflect the similarity of two pairmates relative to their similarity to other scenes from the same condition.

To test the relationship between scene–object memory and scene pair difference score, we used mixed-effects logistic regression. We relied on this method because it allows us to model behaviour as a function of several predictors, accounts for the correlations inherent in repeated measures, and handles unequal bins size and missing data gracefully. We evaluated each subject's scene-object memory behaviour separately for each scene pair in the Same Face and Different Face conditions and each of the three scene-object test cycles. For each scene-object test cycle, we labelled individual scene pairs as being associated with ‘correct' test performance or ‘interference.' We labelled a scene pair ‘correct' if the subject selected the correct object for both scene pairmates and thus avoided interference between the pairmates' associations. We labelled a scene pair as corresponding to ‘interference' if the subject made either one or two interference errors—that is, by selecting the pairmate foil for one or both of the pairmates (Fig. 1c). All other scene pairs (for example, pairs for which subjects selected nonpairmate foils for both scenes) were excluded from analysis.

### fMRI classification analyses

Pattern classification analyses were performed using LIBLINEAR^52^ L2-regularized logistic regression models from Sci-kit Learn^53^ with the default regularization parameter of 1. Every pattern was *z*-scored across voxels before being submitted to analysis.

For classifier-based face reactivation analyses (Supplementary Figs 6a and 7), a second general linear model analysis was performed on each subject's localizer data, with one regressor assigned to each of the 12 face, 12 scene and 12 object blocks. This yielded 12 face and 12 scene patterns of interest. For each subject, a classifier was trained to discriminate faces from scenes using these 24 training examples. We then used this classifier to predict the category of each of the 72 scenes from the main experiment from their estimated neural patterns. For every scene this yielded a prediction (face or scene) as well as a probability estimate for each category. Face evidence was defined as the logit-transformed probability of the face category.

### Regions of interest

ROIs were produced for each subject in native space using a combination of automatic segmentation and voxel selection from the localizer data. We used Freesurfer to automatically define bilateral anatomical ROIs in each subject's native space, including the hippocampus, medial ventral temporal cortex (parahippocampal gyrus and collateral sulcus), and early visual cortex (retinotopic area V1). We manually ensured that hippocampal segmentation was successful. Then, we used data from each subject's visual category localizer to further select scene-preferring voxels (scenes>faces and objects) from the hippocampus (*z*>1) and medial ventral temporal cortex (*z*>3). This yielded a scene-preferring hippocampal ROI (mean=128 voxels) and a PPA ROI (mean=271 voxels) for each subject. We chose a lower threshold in the hippocampus than in ventral temporal cortex because the mean of the scene preference distribution in the hippocampus is lower, and a lower threshold was necessary to yield enough voxels to analyse.

### Statistics

Repeated measures ANOVAs and two-tailed paired *t*-tests were used to assess differences in scene pair difference score across conditions and ROIs. Normality and sphericity of data were confirmed using Shapiro-Wilk and Mauchly's tests. In the case of normality violations, nonparametric Wilcoxon signed-rank tests with continuity correction were used in place of *t*-tests and Friedman's test in place of one-way repeated measures ANOVA. Mixed-effects logistic regression models were used to assess the relationship between scene pair difference score and subsequent scene–object learning, and were implemented in the lme4 package for R (http://lme4.r-forge.r-project.org). All models were constructed with random intercepts for each subject and the maximal random slope structure for within-subject factors^54^. The significance of predictors was assessed using likelihood ratio tests on nested models. Parameter estimates and s.e. for the predictors are also reported.

## Additional information

**How to cite this article:** Favila, S. E. *et al*. Experience-dependent hippocampal pattern differentiation prevents interference during subsequent learning. *Nat. Commun.* 7:11066 doi: 10.1038/ncomms11066 (2016).

## Supplementary Material

Supplementary Information Supplementary Figures 1-7 and Supplementary References
