# Supplementary material for: Experience-dependent hippocampal pattern differentiation prevents interference during subsequent learning
Source: Nat Commun. 2016 Mar 24;7:11066. doi: 10.1038/ncomms11066 (PMC4820837; doi:10.1038/ncomms11066)
Supplement: Supplementary Information — Supplementary Figures 1-7 and Supplementary References [file ncomms11066-s1.pdf]

Supplementary Figures

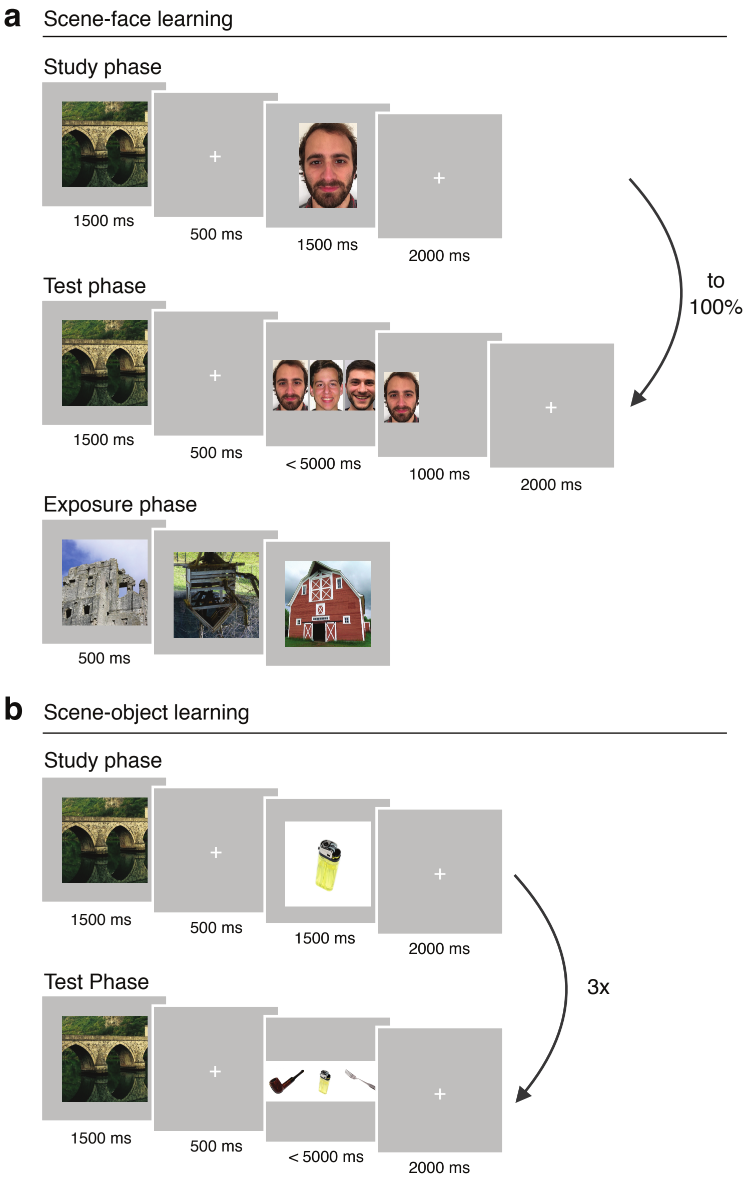

Supplementary Figure 1: Associative learning procedures. (**a**) On day 1 and at the start of day 2, subjects learned scene-face associations for the Different Face and Same Face conditions through interleaved study and test blocks. Each study trial began with the presentation of a scene image followed by the associated face image after a short delay. Each test trial began with presentation of a scene cue followed by a set of three faces—the target face and two foils that had been paired with other scenes. In the Different Face condition, one foil was the face associated with the cue's pairmate ('pairmate foil'). Subjects were instructed to select the target face via button press. Feedback was provided after every trial by showing the target face alone. Subjects completed a total of at least six test trials for each scene and continued until 100% accuracy was achieved. In a separate exposure task, all scenes (including No Face scenes) were viewed repeatedly while subjects detected inverted images. (**b**) Subjects learned scene-object associations on day 2 (following fMRI scanning) through three interleaved study and test blocks following a procedure similar to scene-face learning. Foils during test trials always consisted of the pairmate foil ('interference error') and one nonpairmate foil ('other error'). Feedback was not provided at the end of scene-object test trials. See also Figure 1 and Methods.

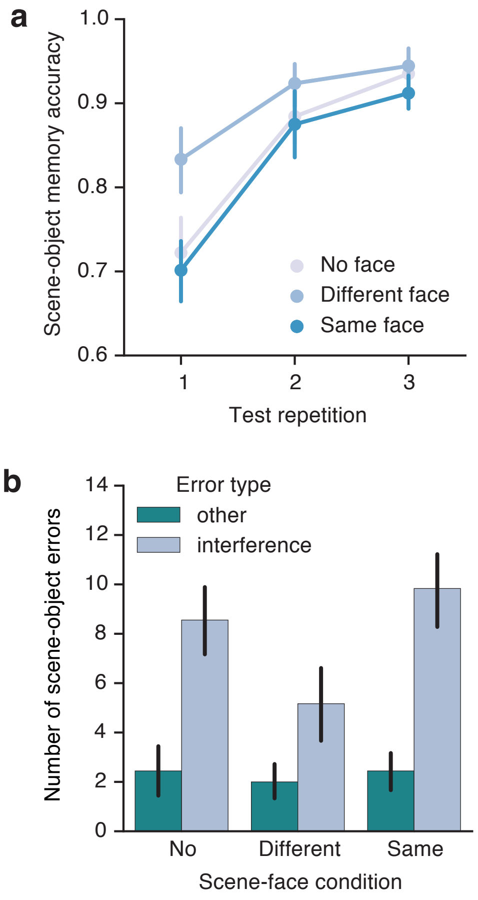

Supplementary Figure 2: Scene-object memory performance. (**a**) Accuracy on scene-object memory test trials is plotted by scene-face condition and scene-object test repetition. There was a main effect of scene-face condition on scene-object memory accuracy (χ^2_2 = 17.41, $P < 0.001$), such that subjects performed best in the Different Face condition. There was also a main effect of test repetition with performance increasing across repetitions (χ^2_1 = 33.81, $P < 0.001$). (**b**) Errors made during scene-object test trials either reflected 'interference errors,' where subjects selected the object that had been associated with the test scene's pairmate, or 'other errors' where subjects selected an object that had been associated with a nonpairmate. Interference errors were much more common than 'other errors' ($F_{1,17}$ = 33.12, $P < 0.001$) confirming that pairmate similarity produced memory interference. Plotted data represent mean ± SEM across subjects.

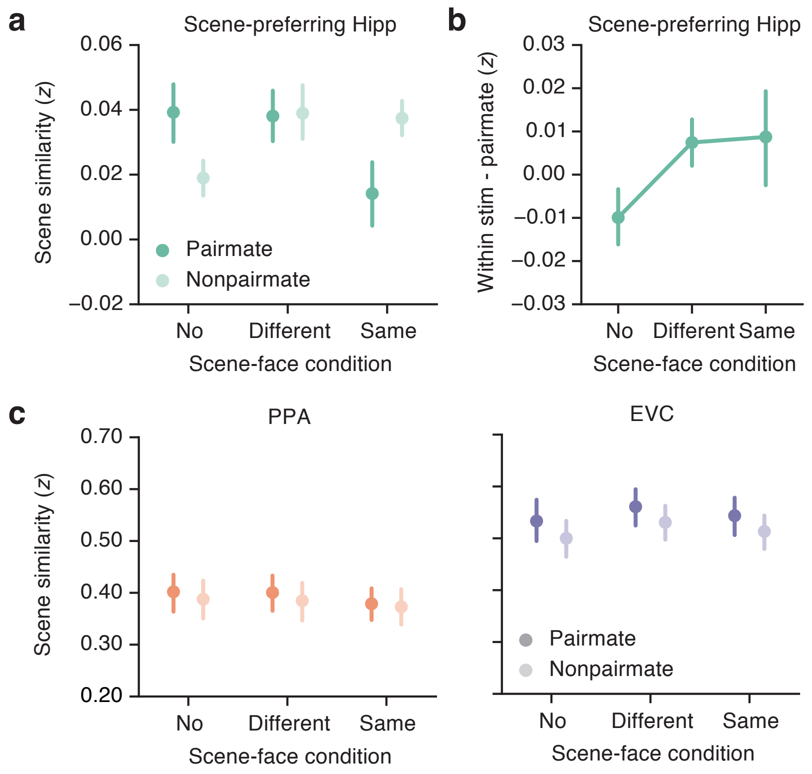

Supplementary Figure 3: Learning-related changes in pairmate and nonpairmate similarity. (**a**) In HIPP, there was a marginally significant main effect of scene-face condition (No Face, Different Face, Same Face) on pairmate similarity ($F_{2,34}$ = 2.75, P = 0.078) and a significant effect on (within-condition) nonpairmate similarity ($F_{2,34}$ = 6.43, P = 0.0043) in scene-preferring hippocampal voxels. (**b**) In HIPP, the difference between within stimulus similarity (similarity between ‘barn 1’ and ‘barn 1’) and pairmate similarity (similarity between ‘barn 1’ and ‘barn 2’) was significantly higher in Different and Same Face conditions relative to the No Face condition in scene-preferring hippocampal voxels [t_{16} = 2.36, P = 0.016; one-tailed; one subject removed due to outlier score (z = -3.0) in the Different face condition]. The difference between within stimulus similarity and pairmate similarity was marginally greater than zero in the Different and Same Face conditions [t_{16} = 1.47, P = 0.08; one-tailed; outlier removed]. To perform these analyses, we split our fMRI data into odd and even runs and estimated the neural pattern of response to each scene in each half of the data using separate general linear models. Within stimulus similarity values represent the Fisher z-transformed Pearson correlation between the pattern corresponding to a stimulus in half 1 and the pattern corresponding to the same stimulus in half 2 (e.g., ‘barn 1, half 1’ vs. ‘barn 1, half 2’). Pairmate similarity values represent the Fisher z-transformed correlation between pairmates across halves of the data (e.g., ‘barn 1, half 1’ vs. ‘barn 2, half 2’). (**c**) In PPA and EVC, there was no main effect of scene-face condition on either pairmate similarity or nonpairmate similarity (all $P > 0.10$). Plotted data represent mean ± SEM across subjects.

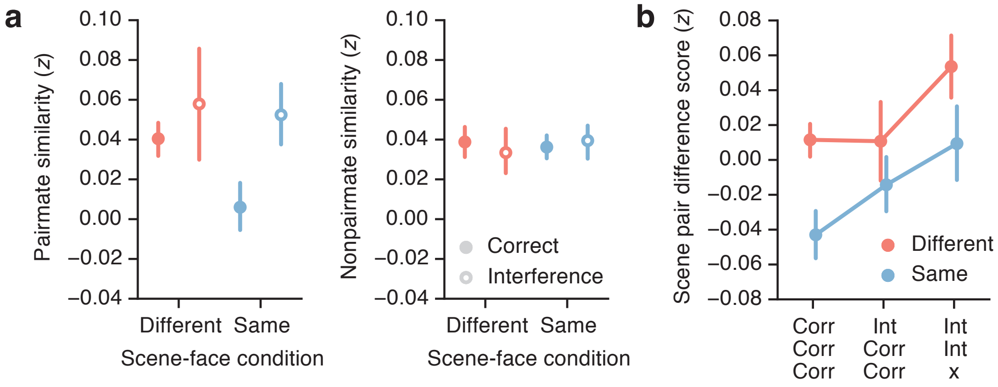

Supplementary Figure 4: Learning-driven decreases in representational overlap prevent interference during future learning. (**a**) In a logistic regression analysis complementary to the one reported in the main results (see Fig. 3a), lower pairmate similarity in the Same Face and Different Face conditions predicted resistance to interference during scene-object learning (χ^2_1 = 4.85, P = 0.028). The only difference between this analysis and that described in Figure 3a is that pairmate similarity was used here instead of pairmate – nonpairmate similarity. We performed the same analysis using nonpairmate similarities. Nonpairmate similarities in the Same Face and Different Face conditions did not predict subsequent interference (χ^2_1 = 0.59, P = 0.44). As a visualization of these results, separate mean pairmate and nonpairmate correlations are plotted as a function of scene-face condition and subsequent scene-object memory (averaged over scene-object test repetition); these can be compared to Figure 3b. (**b**) Scene pair difference scores in the Different Face and Same Face conditions are plotted as a function of accuracy trajectories across test repetitions of scene-object learning. Scene pairs were divided into the following bins corresponding to accuracy on test repetitions 1, 2, and 3, respectively: 1) Correct, Correct, Correct; 2) Interference, Correct, Correct; 3) Interference, Interference, Correct/Interference ('x'). 'Interference' in this case referred to either 1 or 2 interference errors for that scene pair. Because scene-object memory performance was high and scene pairs that followed other accuracy trajectories were excluded, this analysis is underpowered, particularly in the final bin (4 subjects with observations in the Different Face condition and 8 subjects with observations in the Same Face condition). Though there is no significant main effect of scene-object memory trajectory on scene pair difference score (χ^2_2 = 2.62, P = 0.27), we show the results of this analysis because it is qualitatively consistent with the idea that higher representational overlap leads to more persistent interference errors. Plotted data represent mean ± SEM across subjects.

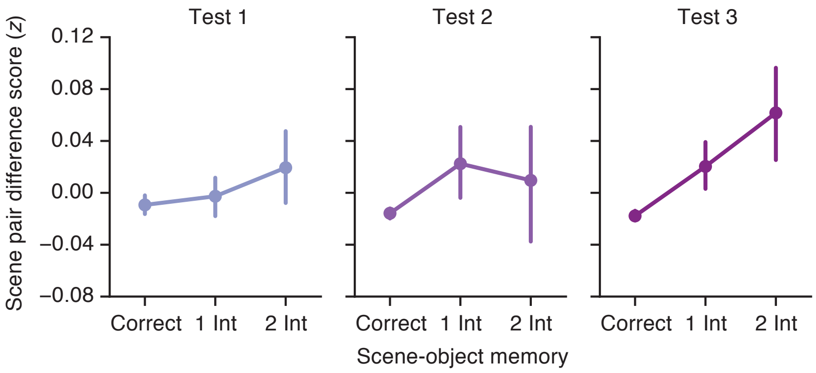

Supplementary Figure 5: Learning-driven decreases in representational overlap as a function of number of interference errors. Scene pair difference scores (averaged across the Different Face and Same Face conditions) are plotted as a function of the number of interference errors per scene pair during each test repetition of scene-object learning. For each test repetition, scene pairs were divided into three bins: subsequently correct (0 interference errors), 1 interference error, or 2 interference errors. Though splitting scene pairs into 1 and 2 interference error groups resulted in low bin sizes—particularly for the 2 interference error bin—we investigated whether there were stepwise differences in scene pair difference score between each bin (i.e., 0 < 1, and 1 < 2), with the prediction that scene pair difference scores should be highest (more representational overlap) in bins with more interference errors. To this end, we performed two separate logistic regression analyses. In the first model, we tested whether scene pair difference score predicted correct versus 1 interference error outcomes on the scene-object memory test. In the second model, we tested whether scene pair difference score predicted 1 interference error versus 2 interference error outcomes. These models were otherwise identical to the one we report in the main results and Figure 3. Although the data for both analyses were numerically consistent with higher representational overlap being associated with more interference errors, the main effect of scene pair difference score fell short of significance in each model (Correct versus 1 interference error: χ^2_1 = 2.08, *P* = 0.15; 1 versus 2 interference error: χ^2_1 = 1.75, *P* = 0.19). For the more highly powered comparison, Correct versus 1 interference error, there was a significant interaction between scene pair difference score and test repetition in predicting memory, such that differences in scene pair difference score between correct vs. 1 interference error bins increased with test repetition (χ^2_1 = 4.98, *P* = 0.026). For the comparison of 1 vs. 2 interference errors, although the difference was numerically greatest at the third repetition, the interaction with test repetition was not significant (χ^2_1 = 0.12, *P* = 0.73). Plotted data represent mean ± SEM across subjects.

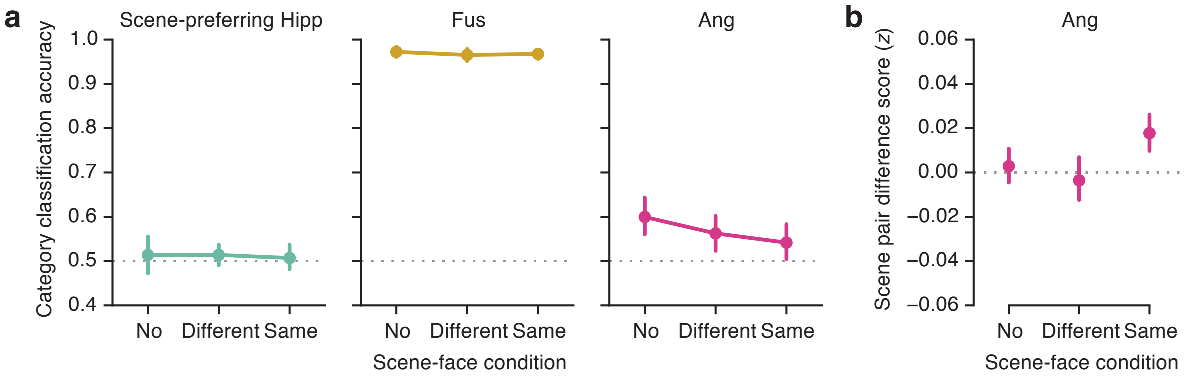

Supplementary Figure 6: Face reactivation in parietal cortex. (**a**) Logistic regression classifiers were trained using each subject's localizer data to discriminate face vs. scene blocks and then tested on the neural patterns corresponding to the 72 scenes from the main experiment (see Methods). Although the correct answer or label was always 'scene,' we predicted that classification accuracy would be lower in the Different Face and Same Face conditions relative to the No Face condition if viewing scenes from these conditions elicited face reactivation. In scene-preferring hippocampal voxels, classification accuracy was not above chance in any of the conditions (all $P > 0.50$), consistent with prior evidence that the hippocampus is relatively insensitive to category-level information despite being important for differentiating individual stimuli[1]. In fusiform gyrus (Fus), accuracy was well above chance in all conditions (all $P < 0.001$), but was not lower for Same Face and Different Face scene pairs than No Face scene pairs ($W_{17} = 13.0$, $P = 0.53$). In angular gyrus (Ang), accuracy was above chance in the No Face condition ($W_{17} = 138.5$, $P = 0.022$), but not in the Different Face condition ($t_{17} = 1.60$, $P = 0.13$) or Same Face condition ($t_{17} = 1.04$, $P = 0.31$). There was a modest, but non-significant, decrease in classification accuracy in the Same/Different Face conditions relative to the No Face condition ($t_{17} = -1.33$, $P = 0.20$). Thus, the pattern of classification data in angular gyrus was qualitatively consistent with face reactivation, but only weakly so. (**b**) Scene pair difference scores in angular gyrus were higher in the Same Face condition than the Different Face condition ($t_{17} = 2.33$, $P = 0.032$) and also significantly greater than zero ($t_{17} = 2.21$, $P = 0.041$). This pattern is consistent with the idea that angular gyrus activity patterns reflected reactivation of the specific faces that subjects learned to associate with the scenes[2], but contrasts sharply with the pattern of scene pair difference scores observed in the hippocampus ($F_{2,34} = 7.08$, $P = 0.0027$). Plotted data represent mean ± SEM across subjects.

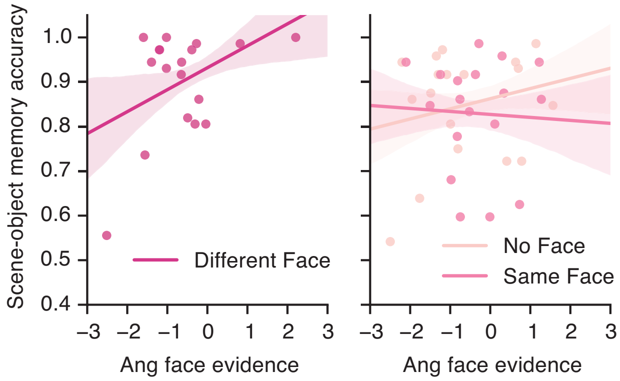

Supplementary Figure 7: Relationship between parietal face reactivation and memory performance. Individual differences in mean classifier evidence for the face category positively correlated with scene-object memory performance in the Different Face condition ($r_{16} = 0.43$, $P = 0.038$; one-tailed). That is, subjects that exhibited higher evidence for face reactivation also performed better on subsequent scene-object learning for scenes that were in the Different Face condition. There was no such relationship within the Same Face or No Face conditions (all $P > 0.10$; one-tailed). These data are consistent with subjects' self-reports. In response to an open-ended question about learning strategies, a majority of subjects (13/18) reported integrating scene-face associations into subsequent scene-object associations, a strategy that would selectively benefit performance in the Different Face condition. Specifically, when scene pairmates had been previously paired with different faces, integrating faces into scene-object associations would add a differentiating element (distinct faces) to the scene-object associations, thereby reducing interference. But when scene pairmates had been associated with the same face or no face, integrating face images into subsequent scene-object learning would be unhelpful or irrelevant, respectively. The fact that many subjects reported integrating face images into subsequent scene-object associations may explain the high levels of behavioral performance we observed in the Different Face condition relative to the Same Face condition. Points represent individual subjects and lines and shading represent linear regression fits and standard errors.

Supplementary References

1. LaRocque, K. F. *et al.* Global Similarity and Pattern Separation in the Human Medial Temporal Lobe Predict Subsequent Memory. *J. Neurosci.* **33,** 5466–5474 (2013).

2. Kuhl, B. A. & Chun, M. M. Successful remembering elicits event-specific activity patterns in lateral parietal cortex. *J. Neurosci.* **34,** 8051–8060 (2014).
